# Data linkage of routinely collected qualitative health data: Using qualitative Patient Reported Experience Measures to understand the impact of digital hospitals on patients

**DOI:** 10.23889/ijpds.v9i5.2509

**Published:** 2024-09-18

**Authors:** Teyl Engstrom, Clair Sullivan, Shirley Thompson, Jacqueline Daly, Jason D. Pole

**Affiliations:** 1 Queensland Digital Health Centre, The University of Queensland; 2 Metro North Health, Queensland Health; 3 Clinical Excellence Queensland, Queensland Health

## Objective

Data linkage and secondary use of routinely collected quantitative health data is ubiquitous. In recent years, the volume and breadth of routinely collected qualitative health data has grown. These data sources provide rich narrative information that could enable a broader understanding of healthcare challenges at scale, however linkage and secondary use of this qualitative health information is not common. This study explores the potential of linkage and secondary use of qualitative patient reported experience measure (PREM) data to understand the impact of digital hospitals on patients.

## Approach

In Queensland, Australia, PREM surveys are distributed to most public hospital patient. Patient responses will be linked to the hospital and ward attended; qualitative comments will be extracted from two large hospitals: a digital hospital and non-digital hospital. Comments will be compared between the hospitals, focusing on factors which relate to experience of a digital hospital, either directly (e.g. computerised workstations), or indirectly (e.g. communication).

## Results & Conclusions

Currently under ethics review – one challenge identified is that deidentification of free-text qualitative data at this scale is not feasible. Ethics committees may not have experience with this type of study and may require education on using qualitative health data at scale.

## Implications

Linkage and secondary use of routinely collected qualitative PREMs data has the potential to reduce participant burden in research, by utilising existing data sources to answer research questions. It can also broaden the scope of research by utilising data collected across multiple hospitals or jurisdictions, without significantly increasing the resources required.

